# LC-MS and GC-TOF-MS metabolomics elucidate quality and metabolite variations of medium-grade Yunyan 87 (C4F) tobacco under stage-specific curing process

**DOI:** 10.3389/fpls.2026.1896995

**Published:** 2026-10-30

**Authors:** Meng Zhao, Chaopeng Song, Jiaen Su, Jun He, Mingdong He, Meixun Fan, Jiangtao Li, Wei Yang, Chunfeng Tao, Hongwei Xiao, Binbin Hu

**Affiliations:** 1 Yunnan Academy of Tobacco Agricultural Sciences, Kunming, China; 2 College of Engineering, China Agricultural University, Beijing, China; 3 Lincang City Tobacco Monopoly Bureau, Lincang, China; 4 Yunnan Daguan Laboratory, Kunming, China

**Keywords:** biosynthetic metabolism, C4F medium-grade tobacco, flue-curing, GC-TOF-MS, LC-MS, metabolomics, Nicotiana tabacum L., stage-specific curing regulation

## Abstract

To investigate the impact of flue-curing parameters on the intrinsic quality of medium grade tobacco (*Nicotiana tabacum* L.) leaves, this study uses C4F grade leaves of the Yunyan 87 variety as the experimental material. Based on the conventional curing process (Z4C), the parameters are adjusted at different stages to establish three optimized treatments: Z4G (yellowing + color-fixing stages), Z4S (color-fixing stage only), and Z4W (stem-drying stage). Metabolomics integrating LC-MS and GC-TOF-MS is employed to decipher the quality changes of C4F leaves under different curing process. The results show that the Z4G process promotes starch degradation and soluble sugar accumulation in tobacco leaves, achieving the optimal performance in aroma content, aroma quality, and overall sensory score. Z4S shortens the curing duration, elevates cinnamic acid content, and suppresses indole synthesis, but yields the lowest comprehensive score. Z4W, through low temperature and prolonged treatment, reduces the degradation of thermosensitive compounds, thereby minimizing the loss of flavonoids, phytol, geraniol, and other substances, while also decreasing the accumulation of alkaloids and their derivatives. This study elucidates the metabolic mechanism by which stage-specific curing regulation of the curing process modulates the quality formation of medium grade flue-cured tobacco, providing a theoretical basis and data support for the precise optimization of the curing protocol for C4F leaves and for enhancing their industrial usability.

## 1 Introduction

Tobacco (*Nicotiana tabacum L.*) is an important industrial cash crop, and the intrinsic quality of its leaves directly determines the industrial value and sensory smoking experience of cigarette products (Lencucha et al., 2023). Curing is the core process that converts harvested fresh leaves into dried products with specific aroma, color, and combustibility (Zong et al., 2022; Chen et al., 2021). The common curing method for flue-cured tobacco is flue-curing, and the traditional curing process is clearly divided into three continuous and interrelated stages: yellowing stages, color-fixing stages, and stem-drying stages (Meng et al., 2024; Chen et al., 2020). These three stages cooperatively regulate a series of complex physiological and biochemical processes, including chlorophyll and starch degradation, the synthesis and transformation of aroma precursors, and the gradual removal of water (Feng et al., 2025; Huang et al., 2026). The process parameters, such as temperature and humidity, at these critical stages alter the pathways of substance conversion and the spatiotemporal accumulation of aroma precursors in tobacco leaves, ultimately determining the comprehensive quality attributes of the cured leaves, including color, aroma, and taste. In China, tobacco leaves are classified into 42 grades, which are further grouped into three categories: superior, medium, and inferior (Liu et al., 2026, Liu et al., 2025). The grading system relies heavily on visual appearance factors, including color, oil content, leaf length, and degree of injury. Among them, middle-grade tobacco leaves account for a large proportion and occupy a vital position in cigarette industrial formulation (Li et al., 2026). C4F tobacco leaves are representative of medium grade tobacco, generally exhibiting low color saturation, loose tissue structure, and a high degree of injury. However, this grading system based on manual experience suffers from an inherent limitation: medium-grade tobacco leaves with nearly identical external features frequently exhibit within quality differentiation in the types, contents and stoichiometric proportions of internal metabolites. Currently, the biochemical essence underlying this quality differentiation and its formation mechanism lack systematic understanding in the academic community (Xiang et al., 2020; Chen et al., 2023).

In recent years, extensive research has been conducted on the regulation of temperature and humidity during tobacco curing. Most of these studies adjust parameters in a single curing stage to investigate the impact of these parameters on tobacco quality or microbial communities (Zhao et al., 2022; Jia et al., 2023; Hu et al., 2021). Other studies, such as the work conducted by Wei et al. (2025a) raise the wet-bulb temperature by 1 °C throughout the entire curing process and prolong the total curing duration to improve the quality of tobacco leaves. Therefore, it is an urgent issue to systematically investigate how alterations in process parameters at different curing stages affect the intrinsic quality of medium grade tobacco leaves that share similar appearance traits. Metabolomics, as an important component of systems biology, offers high-throughput and unbiased detection advantages and has become a powerful tool for dissecting the regulatory mechanisms of plant secondary metabolic networks and the molecular basis of agricultural product quality formation (Oh et al., 2023). Currently, untargeted metabolomics mainly relies on liquid chromatography-mass spectrometry (LC-MS) and gas chromatography-mass spectrometry (GC-MS) technology platforms (Wu et al., 2026). Gas chromatography-time-of-flight mass spectrometry (GC-TOF-MS) is particularly suitable for detecting low-molecular-weight volatile substances, aromatic hydrocarbons, short-chain fatty acids, and thermally stable compounds; moreover, GC-TOF-MS with derivatization effectively covers low-hydrophilicity non-volatile metabolites (He et al., 2025). Current understanding of tobacco leaf curing mechanisms mostly relies on data obtained via LC-MS detection. This technique identifies a limited range of metabolites, which prevents the comprehensive visualization of the overall metabolic network dynamics during curing (Meng et al., 2022; Huang et al., 2026; Wei et al., 2024). In fact, the combined application of LC-MS and derivatization-based GC-TOF-MS greatly improves the coverage depth of metabolic profiles. Nevertheless, research cases adopting such multi-platform integrated analytical strategies remain scarce to date.

To investigate the impact of stage-specific adjustments of curing parameters on the intrinsic quality of medium-grade tobacco leaves, this study uses flue-cured tobacco leaves of the C4F grade as the research object. Based on the traditional three-stage curing process, the key process parameters of the yellowing, color-fixing, and stem-drying stages are systematically optimized. Using metabolomics based on the combination of LC-MS and GC-TOF-MS, this study systematically compares the dynamic differences between the optimized and conventional curing processes in terms of metabolomics profiles, key flavor precursors, and conventional physicochemical indices. This study aims to elucidate the regulatory mechanism of multi-stage curing parameters on the metabolic network of C4F tobacco leaves, thereby providing a solid theoretical basis and data support for improving the industrial usability of medium grade tobacco leaves.

## 2 Materials and methods

### 2.1 Sample preparation

The test materials were C4F grade flue-cured tobacco leaves of Yunyan 87 cultivar, which were planted and cured following standardized local protocols in Cangyuan Wa Autonomous County, Lincang City, Yunnan Province. The experimental field lay between 98°52′–99°43′ E longitude and 23°04′–23°40′ N latitude at an altitude of 1270 m, and all field management practices complied with local standardized cultivation specifications for flue-cured tobacco. Well-cell transplanting was implemented with a plant spacing of 0.5 m and row spacing of 1.2 m, forming a planting density of 1111 plants per mu. Before transplanting, tobacco-specific compound fertilizer (N:P_2_O_5_:K_2_O = 12:12:24) was applied as base fertilizer at a dosage of 20 kg per mu. The first topdressing was conducted 10 days after transplanting, with 20 kg potassium nitrate applied per mu via hole watering 10 cm away from each tobacco plant. The second topdressing was finished 20 days after transplanting, where 18 kg tobacco-specific topdressing fertilizer (N:P_2_O_5_:K_2_O = 15:0:30) mixed with 10 kg potassium sulfate per mu was dissolved and poured in an annular pattern around plants. All tobacco plants were topped uniformly after squaring, with 18–20 functional leaves retained per plant. Leaves reached harvest maturity when 70%–80% of the leaf surface turned yellow-green, the main vein turned white, and leaf trichomes shed. C4F middle leaves with uniform morphological traits and qualified appearance quality were screened in accordance with the national standard GB 2635–1992. All experimental leaves were harvested from the 2nd layer (counting from top to bottom) of a four-layer bulk curing barn, covering the 5th to 10th leaf clips from the exterior inward. In this study, four different curing process were designed, and one independent curing was performed for each process; the conventional local curing procedure served as the control group designated Z4C, while the other three regimes were optimized treatments. Compared with Z4C, Z4G raised both dry-bulb and wet-bulb temperatures during the yellowing stage and lowered the wet-bulb temperature in the color-fixing stage; Z4S reduced the wet-bulb temperature and shortened the curing duration of the color-fixing stage; Z4W decreased the dry-bulb temperature and extended the curing duration of the stem-drying stage. C4F leaves treated by the four curing regimes were labeled Z4C, Z4G, Z4S and Z4W correspondingly. For each curing process, ten intact leaves were randomly collected. For conventional chemical composition analysis, three biological replicates were set up, and for metabolomics analysis, five biological replicates were set up. During sampling, the main vein of each leaf was removed, and the apical and basal quarter sections of each leaf were trimmed off, leaving only the homogeneous central leaf tissue as analytical samples. All samples were wrapped tightly with labeled aluminum foil, snap-frozen immediately in liquid nitrogen after collection, and stored in a −80 °C ultra-low temperature freezer. Detailed temperature and humidity parameters of each curing regime were provided in **Supplementary Table S1**.

### 2.2 Determination of chemical component contents

The contents of water-soluble sugars, total nitrogen, total alkaloids, proteins, and starch were measured following the protocols specified in the Chinese tobacco industry standards YC/T 159-2019, YC/T 161-2002, YC/T 468-2021, YC/T 216-2013, and YC/T 249-2008, respectively. Total free amino acids and total polyphenols were quantified according to the national standards GB/T 8314-2013 and GB/T 8313-2018, respectively. Potassium and chlorine levels were determined using the procedures described in YC/T 217-2007 and YC/T 162-2011, respectively.

### 2.3 Ultrastructure observation

A 1^2^ tissue section was excised from the central part of each leaf sample, positioned 2 cm away from the midvein. Ultrastructural changes were examined by observing ultrathin sections of the palisade tissue using a Hitachi H-600 electron microscope (Kyoto, Japan).

### 2.4 Sensory evaluation

A group of ten certified experts in cigarette sensory quality assessment performed the sensory evaluation of all tobacco samples. To maintain objectivity, each expert independently evaluated every sample three times. The scoring procedure strictly followed the requirements specified in the YC/T 530-2015 standard. Ten key sensory attributes were assessed, each with a predetermined weight: natural tobacco aroma (10 points), aroma content (15 points), aroma quality (15 points), smoke concentration (10 points), sensory irritation (15 points), off-flavors (10 points), smoke cleanliness (10 points), moistness (5 points), residual aftertaste (5 points), and smoking strength (5 points), summing to a maximum total score of 100. The final score for each attribute was determined using the formula ${\bar{\text{X}}}_{\text{i}}=\sum {\text{X}}_{\text{i}}\text{/n}$, where ${\bar{\text{X}}}_{\text{i}}$ was the mean score, $\sum {\text{X}}_{\text{i}}$ was the total score, and n is the number of participants.

### 2.5 Quasi-targeted metabolomics analysis

Widely targeted metabolomics represented an innovative analytical strategy that integrated the high-throughput screening strength of untargeted metabolomics and the superior accuracy and sensitivity of targeted metabolomics for quantitative measurement. Metabolite qualification and quantification in flue-cured tobacco leaf samples were performed via Multiple Reaction Monitoring (MRM) mode on a SCIEX QTRAP^®^ 6500+ hybrid triple quadrupole-linear ion trap mass spectrometer. The hyphenated analytical system consisted of the aforementioned mass spectrometer coupled with an Exion LC ultra-high-performance liquid chromatography system. Chromatographic separation was achieved using a Waters Xselect HSS T3 column (2.1 × 150 mm, 2.5 μm), and a Thermo ST16R refrigerated centrifuge served as the supporting equipment for sample pretreatment. Mass spectrometry-grade solvents including methanol, formic acid and acetonitrile (Thermo Fisher Scientific) were used throughout the entire experiment, alongside ultrapure water generated by a Merck ultrapure water purification system.

Briefly, 100 mg of tobacco leaf powder fully ground with liquid nitrogen was weighed for each biological sample. A total volume of 10 μL mixed internal standard working solution was accurately spiked into each test sample, with stable isotope-labeled (S)-(−)-malic acid-2,3,3-D3 selected as the internal standard to correct extraction recovery and calibrate quantitative results. Subsequently, 500 μL of pre-cooled 80% aqueous methanol solution containing the internal standard was added to the powder. The mixture was thoroughly vortexed, incubated on ice in darkness for 5 min, and then centrifuged at 15,000 g and 4 °C for 20 min. The supernatant was collected, diluted with mass spectrometry-grade ultrapure water to a final methanol concentration of 53%, and subjected to a second round of centrifugation under identical temperature and relative centrifugal force conditions. The final supernatant was harvested for subsequent instrumental injection. Quality control (QC) pooled samples were prepared by mixing equal volumes of extracts from all tested tobacco samples, while blank control samples were prepared using 53% aqueous methanol solution. QC samples were applied to monitor the stability of the entire instrumental platform throughout the analytical batch. The coefficient of variation (CV) of characteristic peaks for all metabolites detected in QC samples was calculated, and only metabolites with CV ≤ 50% were retained for further analysis. Missing values in the retained data were imputed using the KNN algorithm.

The column temperature was maintained constantly at 50 °C during chromatographic elution. Mobile phase A comprised 0.1% formic acid in water, and mobile phase B comprised 0.1% formic acid in acetonitrile. The gradient elution program was set as follows: 0–2 min, held at 2% B; 2–11 min, linear increase of mobile phase B from 2% to 100%; 11–13 min, held at 100% B for column washing; at 13.1 min, mobile phase B rapidly returned to 2%, and the system was equilibrated until 15 min before the next injection. Mass spectrometric data were acquired under MRM mode with both positive and negative electrospray ionization (ESI) polarities. Unified source parameters were set as follows: curtain gas pressure at 35 psi, collision gas level set to Medium, ion source temperature at 550 °C, and both ion source gas 1 and gas 2 pressures fixed at 60 psi. The ion spray voltage was set to 5500 V for positive ionization mode and −4500 V for negative ionization mode.

Metabolite identification was accomplished by searching against novoDB, an in-house full-standard metabolite database independently constructed by Novogene Co., Ltd. The experimental samples used in this experiment were analyzed and constructed under the same chromatographic and mass spectrometric conditions as the information in the standard database. The chromatographic peak area of the product ion (Q3) was adopted to quantify the relative abundance of each metabolite. Five core parameters were simultaneously verified for compound matching: precursor ion (Q1), product ion (Q3), chromatographic retention time (RT), declustering potential (DP), and collision energy (CE). Only metabolites satisfying matches for all five criteria against the in novoDB authentic standard library were retained for subsequent quantitative analysis. All annotated metabolites met the Level 1 identification confidence criteria defined by the Metabolomics Standards Initiative (MSI). Raw MS data were processed for peak picking, peak integration and preliminary pretreatment using SCIEX OS V1.4 software. The built-in peak filtering criteria were defined as minimum peak height = 500, signal-to-noise ratio (S/N) ≥ 5, smoothing factor = 1, and retention time tolerance = 0.3 min. The integrated peak area of each compound exported from the software reflected its relative quantitative level. All qualitative matching information and quantitative peak area data were exported collectively for downstream statistical analyses. Following metabolite identification, the kyoto encyclopedia of genes and genomes (KEGG) pathway database (https://www.genome.jp/kegg/pathway.html), human metabolome database (HMDB) (https://hmdb.ca/metabolites) and LIPID MAPS database (http://www.lipidmaps.org) were jointly used to complete metabolite chemical classification, structural annotation and metabolic pathway assignment.

### 2.6 GC-TOF-MS untargeted metabolomics analysis

GC-TOF-MS analysis was performed using a LECO Pegasus BT mass spectrometer coupled with an Agilent 7890B gas chromatograph. The system was equipped with a Xiangyi H1850-R refrigerated centrifuge, a Xinzhi SCIENTZ-48 tissue grinder, and an Eppendorf 5305 vacuum concentrator. The reagents used included methanol, acetonitrile, and isopropanol (purity ≥99.0%) from Thermo, methoxyamine pyridine (98.0%) from Sigma, BSTFA (>97%) from TCI, and ultrapure water from Millipore, all of which met the required purity specifications for the experiments.

A sample of 50 mg (± 5%) was weighed and mixed with 0.5 mL of a pre-cooled (–20 °C) acetonitrile:isopropanol:water (3:3:2, v/v/v) solution along with 2 mm zirconium beads. The mixture was shaken at 30 Hz using a tissue grinder, followed by ultrasonication in an ice-water bath. An equal volume of the same solvent mixture was then added, and ultrasonication was repeated. Subsequently, the mixture was centrifuged at 12,000 rpm for 2 min, and 500 μL of the supernatant was collected and concentrated to dryness under vacuum. Then, 80 μL of methoxyamine pyridine solution (20 mg/mL) was added, and the mixture was incubated at 60 °C for 60 min. After that, 100 μL of BSTFA-TMCS (99:1) derivatization reagent was added, followed by incubation at 70 °C for 90 min. Finally, the mixture was centrifuged at 14,000 rpm for 3 min, and the supernatant was collected for analysis. QC samples were prepared by pooling equal amounts of all experimental samples and subjected to the same pretreatment procedure, serving to monitor the stability of the chromatography-mass spectrometry system.

The GC-TOF-MS detection conditions were as follows: an Agilent DB-5MS capillary column (30 m × 250 μm i.d., 0.25 μm) was used, with an injection volume of 1 μL, a split ratio of 1:10, and helium as the carrier gas. The temperatures of the injector, transfer line, and ion source were set to 280 °C, 320 °C, and 230 °C, respectively. The column oven program was as follows: the temperature was held at 50 °C for 0.5 min, then ramped up to 320 °C at 15 °C/min and held for 9 min. The ionization voltage was set to –70 V, the scan rate was 10 spectra/s, and the solvent delay was 3 min. All raw mass spectrometry data were processed using the Chroma TOF software (V43, 1ECO) for spectral deconvolution and peak integration. The area of each characteristic peak was used to represent the relative quantitative value of the corresponding compound, and total peak area normalization was subsequently performed to calibrate the quantitative results and obtain final quantitative data for all detected metabolites. The key deconvolution parameters were set as follows: signal-to-noise ratio threshold of 10, peak width of 3–4 s, mass tolerance of 0.2 Da, and smoothing factor of 3. Metabolite identification was accomplished by matching the fragment ions and collision energy information of detected compounds with the reference data in the LECO-Fiehn Rtx5 database. In order to cover the metabolites within the tobacco leaf system as much as possible, metabolites with a similarity score > 200 were retained for subsequent analysis. Missing values were imputed using the minimum peak area detected for that metabolite across all samples (including QC samples) plus a random seed. Specifically, the smallest non-zero value in that row was first identified, and a random decimal between 0 and 1 was then added; this value was ultimately used as the quantitative value in the delivered results. Finally, the identified metabolites were annotated using the KEGG, HMDB, and LIPID MAPS database.

### 2.7 Statistical analysis

Statistical evaluation of the data was conducted using IBM SPSS Statistics 27.0 (IBM Corporation, Armonk, NY, USA) software, where one-way analysis of variance (ANOVA) was applied, followed by Duncan’s test for multiple comparisons. Graphical representations, including bar charts and radar plots, were generated using Origin 2021 (OriginLab, USA). Visualization of Venn diagrams, combined radar plots, and principal component analysis (PCA) results was performed through the Metware Cloud online platform (https://cloud.metware.cn). Metabolite annotation was carried out based on the KEGG compound database (http://www.kegg.jp/kegg/compound/), and the resulting annotations were subsequently mapped to the KEGG pathway database (http://www.kegg.jp/kegg/pathway.html) to retrieve relevant pathway information. For pathways involving significantly differential metabolites, metabolite set enrichment analysis (MSEA) was further performed, with statistical significance determined using p-values derived from the hypergeometric distribution test.

## 3 Results

### 3.1 Morphological characteristics, ultrastructure, chemical composition, and sensory evaluation of tobacco leaves

Digital images of tobacco leaf samples before harvest and before curing are displayed in **Figures 1I–K**. The ultrastructure and external appearance of C4F tobacco leaves obtained from different curing processes are presented in **Figure 1A**. Leaves of this grade exhibited unsatisfactory color and a high degree of injury, which served as the main criteria for classifying them as medium grade tobacco. Ultrastructural observation revealed that the Z4C samples still contained numerous residual starch granules (marked with red circles), indicating incomplete degradation. In contrast, the Z4S and Z4G samples showed noticeably fewer starch residues, reflecting improved degradation efficiency. Quantitative analysis further confirmed (**Figure 1C**) that starch content was lowest in Z4G, followed by Z4S. Total sugar and reducing sugar contents (**Figure 1B**) displayed the opposite trend, suggesting that starch was effectively converted into reducing sugars. Protein content was relatively low in Z4G and Z4W, while total amino acid content was higher in these groups (**Figure 1E**), indicating that proteins were broken down into amino acids. Total nitrogen and total alkaloid contents (**Figure 1D**), which are generally associated with smoke strength and irritation, were both found at lower levels in the Z4G and Z4S treatment groups. Potassium and chlorine contents markedly influenced the combustion characteristics of tobacco leaves. Among the treatments, Z4G exhibited the highest potassium level and the lowest chlorine level, whereas Z4S had the highest chlorine content (**Figure 1F**). High-quality flue-cured tobacco typically requires a potassium content ranging from 2.0% to 3.5%; in this study, all treatment groups except Z4C met this criterion. Compared with fresh tobacco leaves (FL), the contents of reducing sugars and total sugars increased significantly after curing, which could be attributed to the hydrolysis of starch granules into small-molecule sugars (**Figure 1C**). In addition, continuous protein degradation induced simultaneous elevation of amino acid concentrations at the end of curing. The total polyphenols, total nitrogen and total alkaloids all exhibited higher levels relative to fresh leaves.

**Figure 1:**
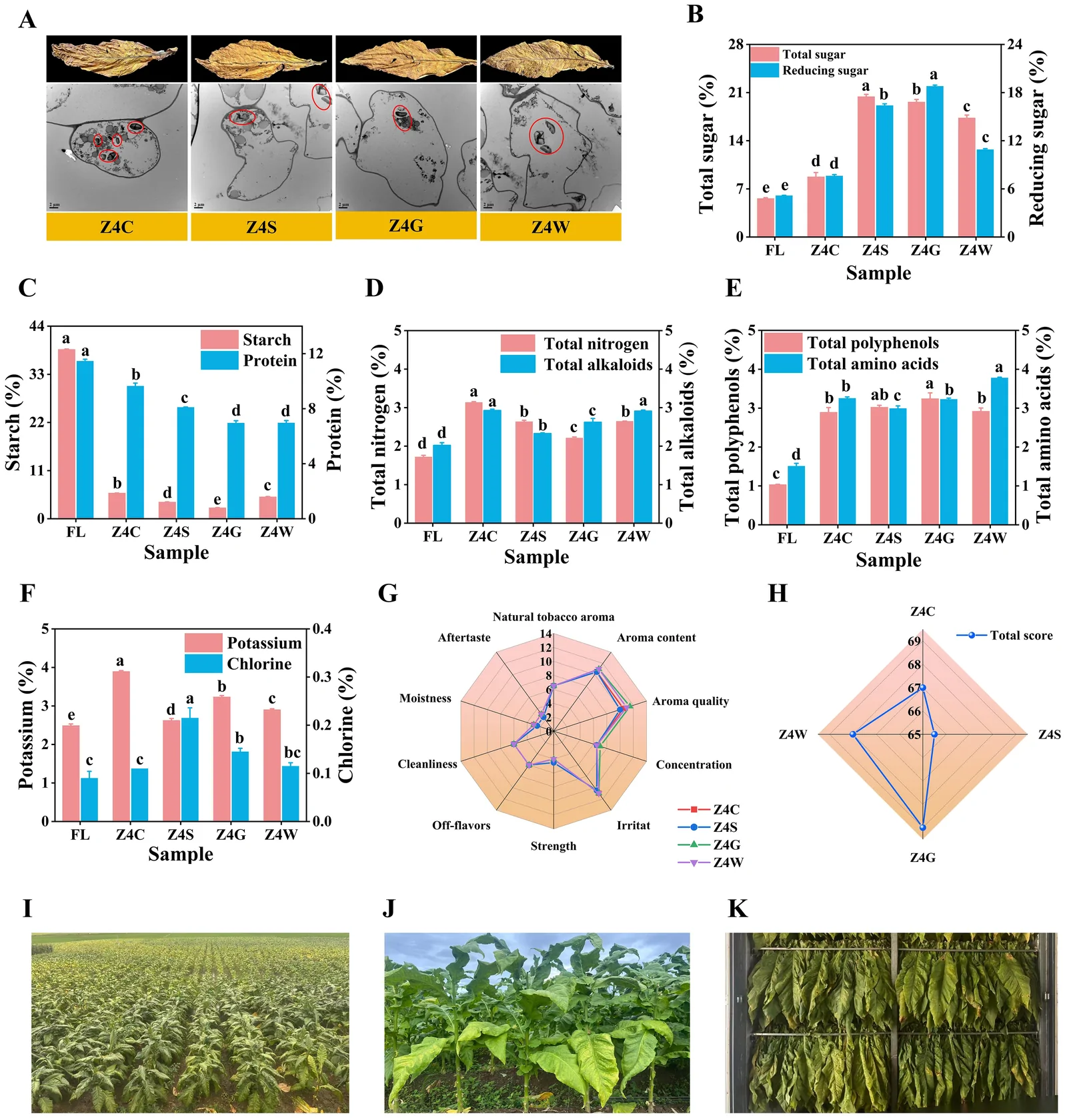
**(A)**, Appearance and chloroplast ultrastructure changes of tobacco leaves; **(B)**, Total sugar and reducing sugar contents; **(C)**, Starch and protein contents; **(D)**, Total nitrogen and total alkaloid contents; **(E)**, Total polyphenol and total amino acid contents; **(F)**, Potassium and chlorine contents; **(G)**, Radar plot of characteristic sensory evaluation scores; **(H)**, Radar plot of overall sensory scores. **(I, J)**, Images of field-grown tobacco plants prior to sample collection; **(K)**, Images of tobacco leaf samples after harvest and before curing. All physiological and chemical indexes were measured with three biological replicates per treatment. For the bar charts in images **1B–F**, statistical analysis was performed using one-way ANOVA followed by Duncan’s multiple range test. Error bars in bar charts represent mean ± standard error (SE). Different lowercase letters denote statistically significant differences between treatments at *P < 0.05*.

Sensory evaluation results are shown in **Figures 1G, H**. For the three attributes of natural tobacco aroma, cleanliness, and off-flavors, all four groups received identical scores of 6.5, 6.0, and 6.0, respectively, suggesting that the different curing processes had a limited impact on these basic sensory properties. However, in terms of aroma content, aroma quality, and smoke concentration, the Z4G samples scored the highest, indicating superior aroma characteristics. Regarding the overall sensory score, Z4G achieved 69 points, which was 2 points higher than that of Z4C (67 points), whereas Z4S obtained the lowest overall score of only 65.5 points. These results indicated that the Z4G process achieved the best overall sensory performance, whereas Z4S and Z4W showed trade‑offs between curing efficiency and quality attributes. To understand the metabolic basis of these differences, we next performed untargeted metabolomics using LC‑MS and GC‑TOF‑MS.

### 3.2 Quasi-targeted metabolomics analysis of tobacco leaves

A total of 1,944 metabolites were identified across all samples using quasi-targeted metabolomics (**Supplementary Table S2**). These metabolites were predominantly classified into the following categories: phenylpropanoids and polyketides (23.12%), organoheterocyclic compounds (18.22%), organic acids and derivatives (17.82%), lipids and lipid-like molecules (15.42%), benzenoids (7.71%), and organic oxygen compounds (5.51%) (**Figure 2B**). Principal component analysis (PCA) score plots (**Figure 2A**) revealed that the first two principal components accounted for 22.06% and 10.69% of the total variance, respectively, with no clear separation observed among the different treatment groups. In contrast, QC samples exhibited excellent clustering performance, and their total ion current (TIC) chromatograms are presented in **Supplementary Figures S1A, B**. Pearson correlation analysis among QC samples further demonstrated high stability and reproducibility throughout metabolite extraction and detection procedures (**Supplementary Figure S1C**). As a unsupervised multivariate statistical method, PCA failed to accurately identify intergroup differences among samples from different treatments. To further precisely discriminate the discrepancies between treatment groups, we adopted the supervised partial least squares-discriminant analysis (PLS-DA) model to visually resolve the grouping profiles of samples, with the results presented in **Supplementary Figure S2**. Within the preset confidence interval, obvious spatial separation was observed among clusters of different groups. The R²Y values for comparisons between Z4C and Z4G, Z4C and Z4S, Z4C and Z4W were 0.99, 0.97 and 1.00, respectively; the corresponding Q²Y values were 0.64, 0.55 and 0.91. The permutation test revealed that all Q² values generated after random permutation were lower than the Q² value of the original model, and the intercept of the Q² regression line on the vertical axis was less than or equal to 0, which demonstrated that the constructed model had no overfitting problem.

**Figure 2:**
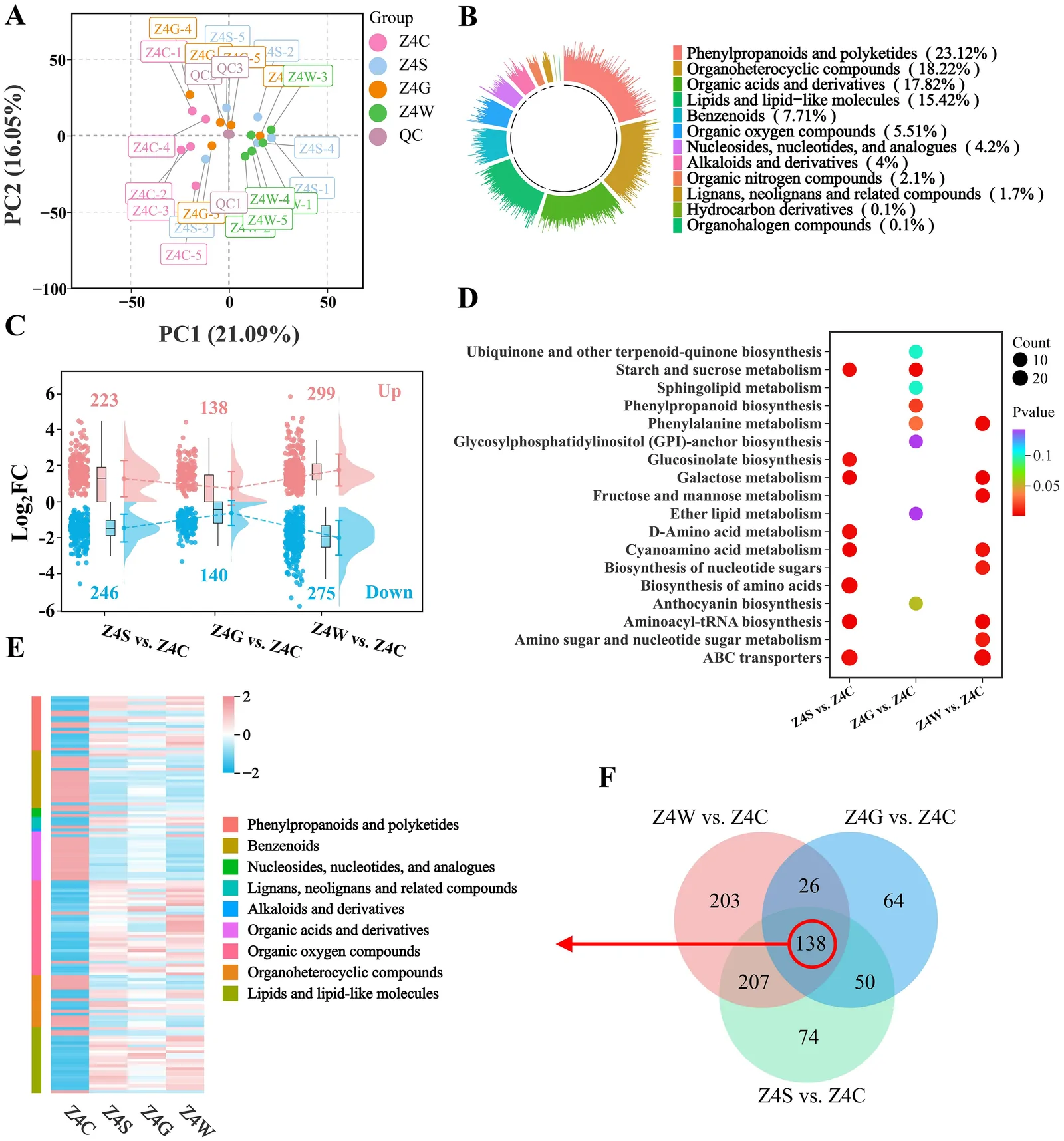
**(A)**, PCA score plot; **(B)**, Classification proportion of all metabolites; **(C)**, Volcano plot of upregulated and downregulated metabolites between comparison groups; **(D)**, Combined enrichment bubble plot among comparison groups; **(E)**, Classification heatmap of shared DAMs between comparison groups; **(F)**, Venn diagram among comparison groups. The heatmaps are generated based on the average metabolite peak areas obtained from five biological replicates of each group.

Differentially accumulated metabolites (DAMs) were screened using the following criteria: Variable importance in projection(VIP) > 1.0, fold change (FC) > 1.2 or FC < 0.833, and *P < 0.05* from univariate two-tailed independent-sample Student’s t-test, the statistical results for all metabolites in each comparison were presented in **Supplementary Table S6**. As shown in **Figure 2C**, a total of 233 upregulated and 246 downregulated DAMs were identified in the Z4S vs. Z4C comparison. In the Z4G vs. Z4C comparison, 138 upregulated and 140 downregulated DAMs were detected. For the Z4W vs. Z4C comparison, 299 upregulated and 275 downregulated DAMs were found. A Venn diagram (**Figure 2F**) illustrated the numbers of shared and unique DAMs among the comparison groups, revealing that 138 DAMs were common to all three comparisons (**Supplementary Table S3**). A classification heatmap (**Figure 2E**) indicated that these shared DAMs mainly belonged to the categories of organic oxygen compounds, phenylpropanoids and polyketides, and benzenoids. Furthermore, 74, 64, and 203 unique DAMs were respectively identified in the Z4S vs. Z4C, Z4G vs. Z4C, and Z4W vs. Z4C comparisons (**Supplementary Table S3**), and these unique metabolites were considered key compounds responsible for the metabolic differences between each optimized curing process and the control. KEGG enrichment analysis (**Figure 2D**) demonstrated that the DAMs from all three comparisons were co-enriched in pathways such as starch and sucrose metabolism, phenylpropanoid biosynthesis, phenylalanine metabolism, and glucosinolate biosynthesis. These metabolic pathways are closely associated with tobacco aroma formation and sensory quality, serving as critical metabolic nodes through which curing processes and tobacco grade coordinately regulate leaf quality.

#### 3.2.1 Identified aroma-related DAMs

Based on the 762 DAMs identified, this study further focused on four categories of compounds closely associated with tobacco aroma quality: flavonoids, terpenoids and their derivatives, amino acids, and fatty acids. Among all DAMs, 41 flavonoids were identified (**Supplementary Table S4**). The clustering heatmap (**Figure 3A**) showed that, compared with the Z4C group, the relative contents of most flavonoids in the Z4W treatment group exhibited an increasing trend, while some flavonoids were upregulated in the Z4S group; the magnitude of change in the Z4G group fell between that of Z4C and Z4W. A total of 46 terpenoids and their derivatives were detected (**Figure 3B**, **Supplementary Table S4**). Across the treatment groups, an overall upward trend in these compounds was observed relative to Z4C, with the most pronounced increase occurring in the Z4W group. Twenty-eight amino acid DAMs were identified (**Figure 3C**, **Supplementary Table S4**), and marked differences were found among the curing processes. Their relative expression levels followed a descending order of Z4C > Z4G > Z4S > Z4W. Fatty acid compounds serve as important precursors for volatile aroma components such as aldehydes, alcohols, and ketones. In this study, 22 fatty acid DAMs were detected (**Figure 3D**, **Supplementary Table S4**). Their accumulation pattern indicated that most fatty acids were highly expressed in the Z4C and Z4W groups, whereas overall expression levels were relatively lower in the Z4S and Z4G groups. In summary, different curing processes, by modulating temperature, humidity, and time parameters during the curing procedure, significantly influenced the accumulation characteristics of the above-mentioned key metabolites in tobacco leaves, thereby potentially exerting differential effects on leaf aroma quality.

**Figure 3:**
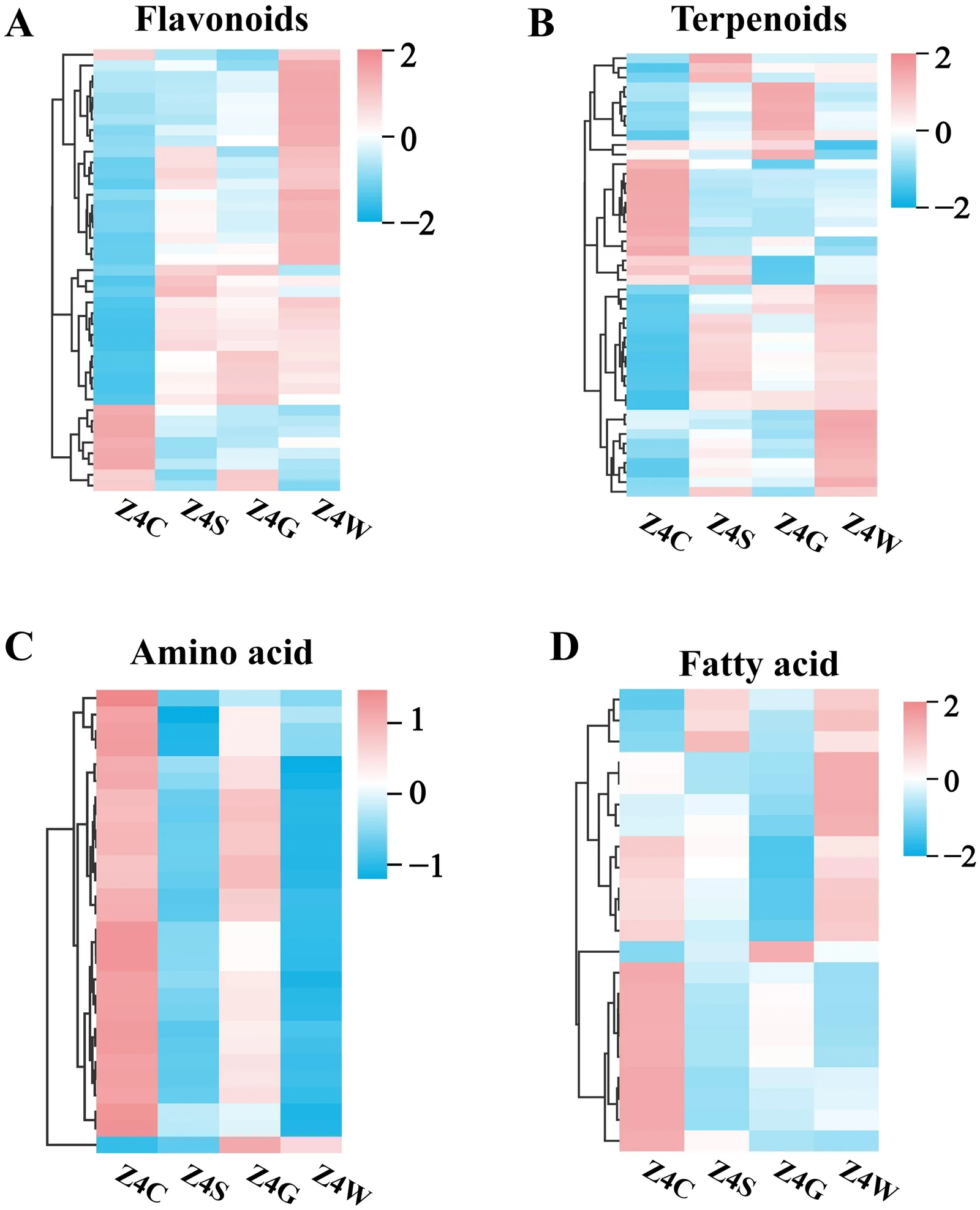
Clustering heatmaps of various classes of DAMs. **(A)**, Flavonoids; **(B)**, Terpenoids and their derivatives; **(C)**, Amino acids; **(D)**, Fatty acids. The heatmaps are generated based on the average metabolite peak areas obtained from five biological replicates of each group.

To further dissect the interactive regulatory networks of core DAMs under different curing process, Pearson correlation analysis is performed on screened DAMs belonging to four quality-related categories: amino acids, fatty acids, flavonoids and terpenoids. The absolute threshold of Pearson correlation coefficient is set as |r| ≥ 0.9 with a statistical cutoff of *P < 0.05*, and chord diagrams of metabolite correlation are separately constructed for three comparison groups (**Supplementary Figure S4**). The results reveal that positive correlations dominate the metabolite association networks across all three groups, and intra-group interactions within the same metabolite class constitute the primary framework of the whole network. For the comparison of Z4S vs. Z4C, frequent cross-category interactions occur between amino acids and terpenoids, most of which are significantly positive correlations. Among them, two terpenoids including Melittoside (Com_1843_neg) and Oleoside 11-methyl ester (Com_1755_neg) exhibit markedly negative correlations with the majority of amino acid components, whereas 1beta-Hydroxyalantolactone (Com_600_pos) and Inulicin (Com_206_pos) show strong positive correlations with multiple amino acids. In addition, the amino acid L-Histidine (Com_1336_neg) establishes a negative regulatory relationship with the flavonoid Neoisoliquiritin (Com_228_pos). The metabolite correlation architecture of the Z4G vs. Z4C group is relatively simple, whose network framework largely relies on synergistic positive correlations inside each metabolite category with limited cross-category crosstalk. Specifically, the flavonoid Complanatuside (Com_244_pos) is positively correlated with three terpenoid metabolites, i.e., Rebaudioside A (Com_1522_neg), Abscisic acid glucose ester (Com_1651_neg) and Geniposide (Com_1501_neg). By contrast, the Z4W vs. Z4C group presents more intricate cross-category interactions; several amino acids including Norvaline (Com_80_pos), Pyroglutamic acid (Com_1284_neg) and Sarcosine (Com_99_pos) are significantly negatively correlated with numerous terpenoids and flavonoids. Collectively, distinct curing process remodel the interactive regulatory networks of core quality-determining metabolites in tobacco leaves.

#### 3.2.2 Analysis of tobacco alkaloids and their derivatives

The relative content variations of eight tobacco alkaloids and their derivatives under different curing processes were further analyzed (**Figure 4A**). Among these, nicotine, anatabine, and anabasine share common biosynthetic pathways. The results showed that the relative content of nicotine was highest in the Z4W group, whereas the levels of anatabine and anabasine were lowest in this group. In the other samples, the trends in nicotine content were generally opposite to those of anatabine and anabasine. 4-(Methylnitrosamino)-1-(3-pyridyl)-1-butanone (NNK), a carcinogenic tobacco specific nitrosamine (TSNA) formed by the nitrosation of nicotine, exhibited its highest relative content in the Z4G group, with an increase of 1.77 times over the Z4C group, and the difference between the two groups was highly significant (*P < 0.01*), whereas the differences between Z4S and Z4W and Z4C were not significant (*P > 0.05*). S-(-)-Cotinine, a characteristic metabolite of nicotine, showed an overall trend opposite to that of nicotine and reached its minimum relative content in the Z4W group. Myosmine, a minor alkaloid in tobacco, displayed relatively higher levels in the Z4W and Z4G groups, with significant differences from the Z4C group (*P<0.01*). Furthermore, the highest relative contents of beta-nicotyrine and cytisinicline were detected in the Z4C and Z4G groups, respectively, while the lowest values for both compounds were found in the Z4W group. Collectively, these results indicated that different curing processes significantly influenced the accumulation patterns of tobacco alkaloids and their derivatives, manifesting as differential distribution of each component across treatment groups.

**Figure 4:**
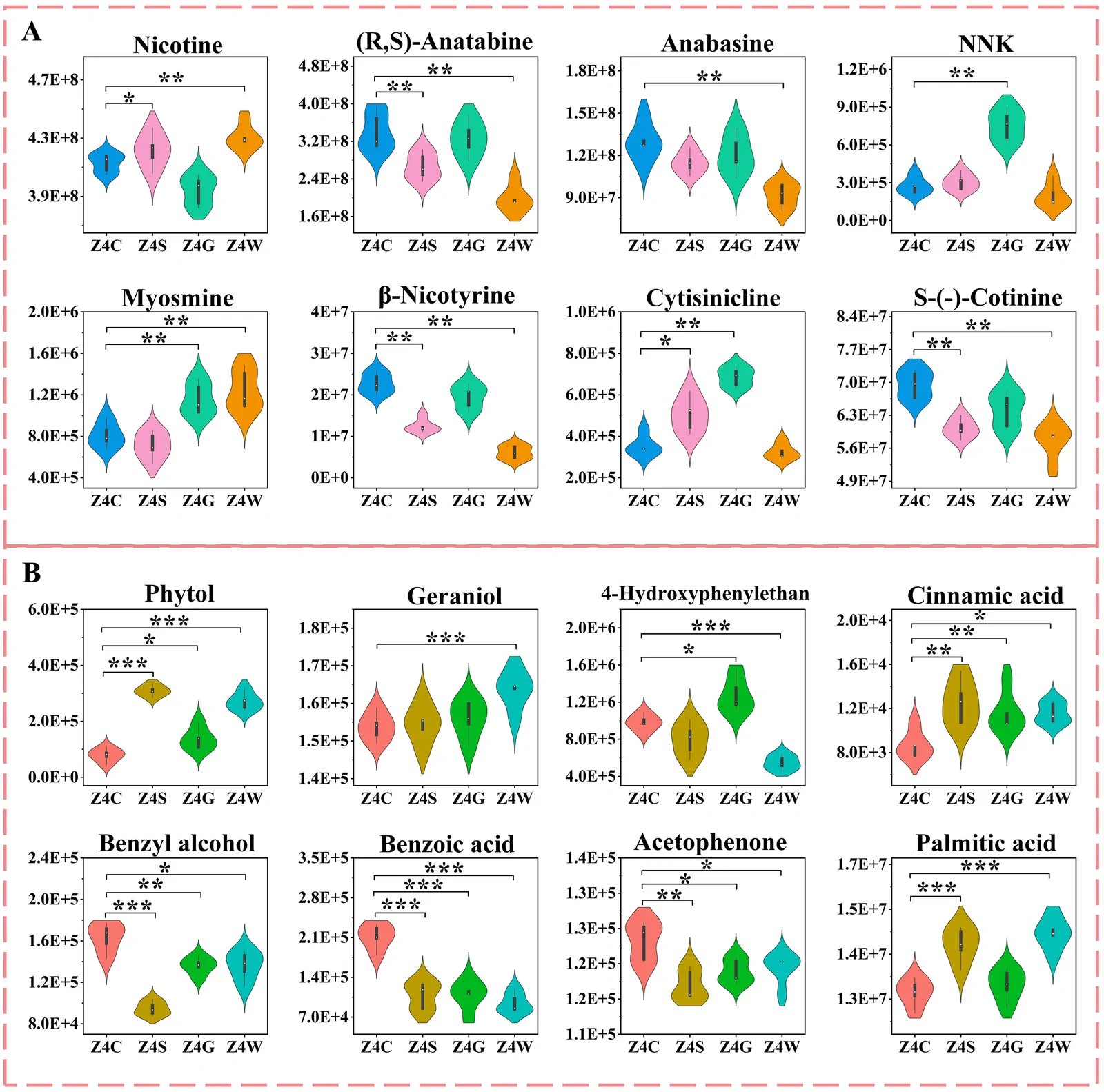
**(A)**, Violin plots showing relative contents of alkaloids and their derivatives identified by LC-MS; **(B)**, Violin plots showing relative contents of key volatile compounds identified by GC-TOF-MS. Violin plots were generated based on the peak area values of five biological replicates for each compound. Inter-group statistical differences were evaluated using independent sample t-test. Asterisks indicate statistically significant differences (*P < 0.05 represents a significant difference; **P < 0.01 represents an extremely significant difference; ***P < 0.001 represents a very highly significant difference).

### 3.3 GC-TOF-MS analysis of tobacco leaves

To achieve a more comprehensive coverage of metabolite species, derivatization followed by GC-TOF-MS analysis was further employed for metabolite identification. A total of 297 metabolites were identified using the GC-TOF-MS platform (**Supplementary Table S5**). Comparison with the LC-MS results revealed that 129 of these metabolites had already been detected by LC-MS, while the GC-TOF-MS platform newly identified 168 metabolites, demonstrating the complementarity of the two analytical platforms (**Figure 5B**). The results of QC samples in the PCA score plot (**Figure 5G**) and pearson correlation analysis (**Supplementary Figure S1D**) fully verified the stability of the analytical system and the reliability of the obtained data. The PLS-DA results revealed obvious spatial separation of sample clusters among all treatment groups, the R²Y values for comparisons between Z4C and Z4G, Z4C and Z4S, and Z4C and Z4W were 0.95, 0.99 and 1.00, respectively; the corresponding Q²Y values were 0.53, 0.49 and 0.88. Permutation validation confirmed that the established models exhibited no overfitting. (**Supplementary Figure S3**). The same criteria (VIP > 1.0, FC > 1.2 or FC < 0.833, and *P < 0.05* from univariate two-tailed independent-sample Student’s t-test) were applied to screen for DAMs, the statistical results for all metabolites in each comparison were presented in **Supplementary Table S7**. As shown in **Figure 5A**, 47 DAMs (14 upregulated and 33 downregulated) were identified in the Z4S vs. Z4C comparison; 14 DAMs (6 upregulated and 8 downregulated) were identified in the Z4G vs. Z4C comparison; and 63 DAMs (18 upregulated and 45 downregulated) were identified in the Z4W vs. Z4C comparison. Further analysis indicated that 6, 8, and 21 unique DAMs were respectively detected in the Z4S vs. Z4C, Z4G vs. Z4C, and Z4W vs. Z4C comparisons. In addition, three common DAMs were identified, namely bis(2-hydroxypropyl)amine (Com_262), 1-kestose (Com_54), and D-erythro-sphingosine (Com_80). KEGG enrichment analysis revealed that the DAMs from all three comparisons were co-enriched in pathways such as tyrosine metabolism, tryptophan metabolism, starch and sucrose metabolism, and the biosynthesis of phenylalanine, tyrosine, and tryptophan (**Figures 5C**).

**Figure 5:**
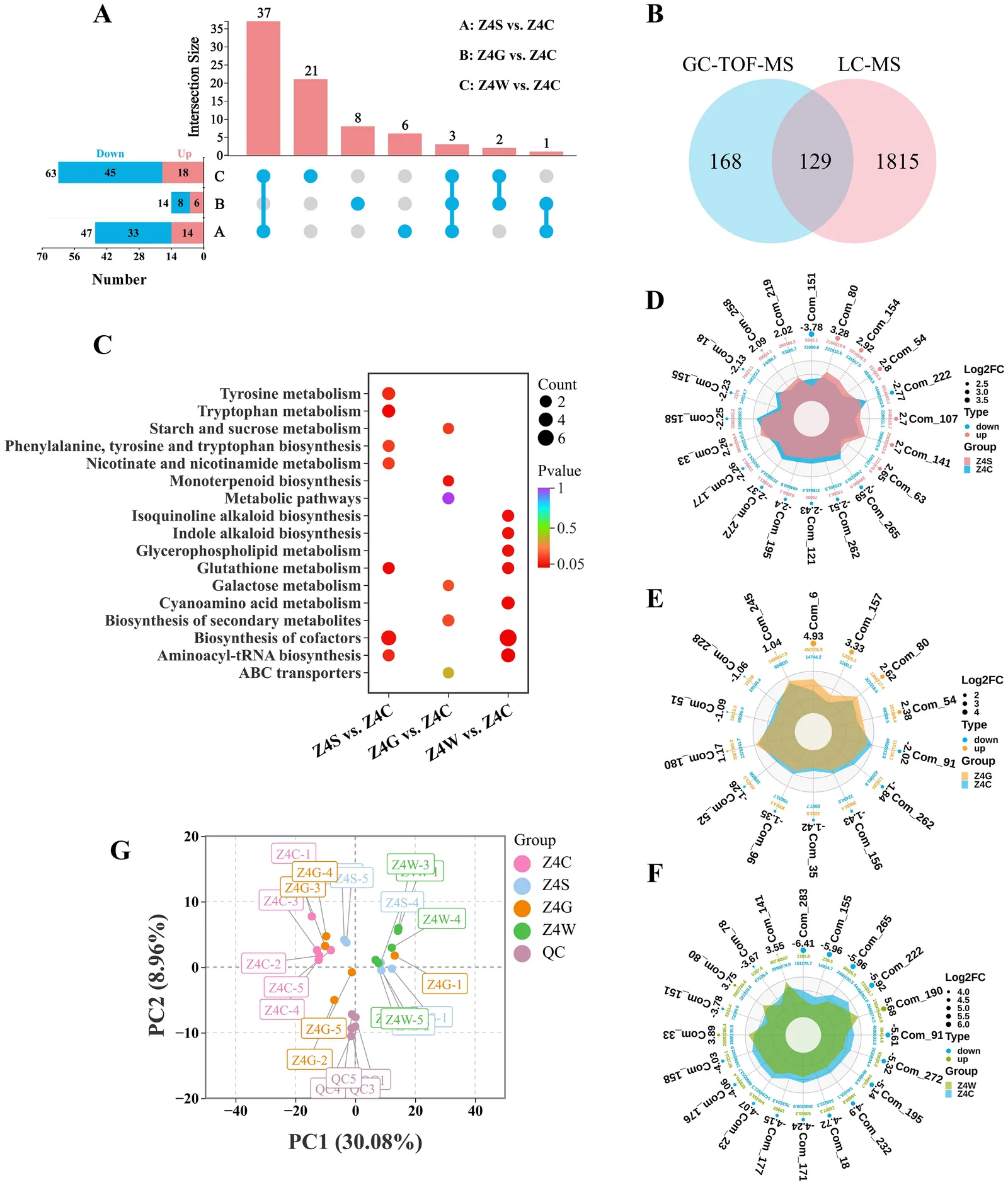
**(A)**, Upset Venn diagram and bar plot of upregulated DAMs for Z4S vs. Z4C, Z4G vs. Z4C and Z4W vs Z4C; Red indicates upregulated differentially accumulated metabolites (DAMs), while blue represents downregulated DAMs. **(B)**, Venn diagram of compounds identified by LC-MS and GC-TOF-MS; **(C)**, Combined enrichment bubble plot among comparison groups; **(D)**, Combined radar plot of the top 20 DAMs ranked by |Log_2_FC| in Z4S vs. Z4C; **(E)**, Combined radar plot of DAMs in Z4G vs. Z4C; **(F)**, Combined radar plot of the top 20 DAMs ranked by |Log_2_FC| in Z4W vs. Z4C.

The combined radar plots in **Figures 5D–F** respectively displayed the top 20 metabolites ranked by |Log_2_FC| in the Z4S vs. Z4C, Z4G vs. Z4C, and Z4W vs. Z4C comparisons. In the Z4S vs. Z4C comparison, the top 20 metabolites consisted of 9 upregulated and 11 downregulated compounds. Among these, the relative levels of several saccharides, including melibiose (Com_33), glucosaminic acid (Com_107), and 3,6-anhydro-D-galactose (Com_141), were increased, whereas the relative contents of amino acids such as glycine (Com_121), aspartic acid (Com_158), lysine (Com_177), and asparagine (Com_222) showed decreasing trends. In the Z4G vs. Z4C comparison, as previously noted, 14 DAMs were identified. Among them, the relative contents of sugars and sugar phosphates, including trehalose-6-phosphate (Com_180), 2-deoxy-D-galactose (Com_228), 1-kestose (Com_54), and sucrose (Com_6), were upregulated. Notably, sucrose exhibited an FC value as high as 30.57, indicating that the sucrose content under this process was substantially higher than that under the conventional process. In the Z4W vs. Z4C comparison, the top 20 metabolites ranked by |Log_2_FC| were predominantly downregulated, with 16 compounds showing reduced levels, most of which were amino acids. These included aspartic acid (Com_158), serine (Com_171), glutamic acid (Com_176), lysine (Com_177), cysteine (Com_18), phenylalanine (Com_91), asparagine (Com_222), and tryptophan (Com_23), all of which were downregulated. Among the upregulated metabolites, glucose (Com_190) stood out with an FC value of 51.31.

#### 3.3.1 Volatile compounds identified by GC-TOF-MS

To investigate the regulatory effects of different curing processes on key volatile aroma components in tobacco, eight volatile organic compounds closely associated with the sensory quality of flue-cured tobacco were selected for analysis (**Figure 4B**). Phytol, a core side-chain degradation product of chlorophyll, can be further converted via dehydration into neophytadiene, which imparts fresh and hay-like notes and contributes importantly to the aroma release and sweetness of flue-cured tobacco. In this study, the relative content of phytol was significantly higher under the Z4S, Z4G, and Z4W processes than under the Z4C treatment, with the highest accumulation levels observed in the Z4S and Z4W groups. Geraniol, a characteristic monoterpene aroma component, imparts fresh floral and sweet notes to tobacco and serves as an important substance for improving the texture of smoke aroma. The results showed that geraniol content was significantly elevated under the Z4W process, whereas no significant differences were found between the other treatments and the control, indicating that a low-temperature, prolonged stem-drying process effectively reduced the thermal degradation loss of such thermosensitive terpenoids. 4-Hydroxyphenylethanol, cinnamic acid, benzyl alcohol, benzoic acid, and acetophenone belong to the class of phenylpropanoid metabolites. These compounds are major sources of floral, fruity, and sweet notes in tobacco and also act as important regulators of smoke irritation and off-notes. Among them, the relative content of 4-hydroxyphenylethanol was significantly higher under the Z4G process than under the other treatments, while it markedly decreased under the Z4W process. Cinnamic acid showed varying degrees of increase across all three optimized processes, with the most pronounced elevation observed in the Z4S treatment. Benzyl alcohol and benzoic acid both exhibited the highest contents in Z4C, and their relative accumulation levels were significantly reduced under the Z4S process. Acetophenone content was highest in the Z4C treatment; the Z4S process significantly lowered its level, whereas no significant differences were detected between the Z4G or Z4W treatments and Z4C. Palmitic acid, a fatty acid that primarily affects the smoothness and mellowness of smoke, is an important component modulating the waxy mouthfeel of smoke. The results demonstrated that both the Z4S and Z4W processes significantly increased the relative content of palmitic acid, with the highest accumulation level occurring in the Z4W treatment, suggesting that this process may be more favorable for improving smoke mellowness.

### 3.4 Analysis of key metabolic pathways in response to different curing process

Based on the KEGG enrichment results obtained from LC-MS and GC-TOF-MS, the starch and sucrose metabolism pathway, as well as the biosynthesis and metabolism pathways of phenylalanine, tyrosine, and tryptophan, were mapped and analyzed. In the starch and sucrose metabolism pathway, compared with the Z4C treatment, the Z4G treatment promoted the conversion and accumulation of sucrose into trehalose and trehalose-6P, while the maltose content also showed an increasing trend. This result indicated that raising the temperature and humidity during the yellowing stage accelerated the rearrangement and transformation of carbohydrates within the tobacco leaves. The shikimate pathway serves as the central route for aromatic amino acid synthesis. In this pathway, quinate was progressively converted to shikimate, which then generated the key node compound chorismate; chorismate was subsequently channeled into the synthesis of tyrosine, phenylalanine, and tryptophan. Notably, the abundances of shikimate and chorismate were lower in the Z4S group, which might have reduced the overall synthesis efficiency of aromatic amino acids. In the tryptophan metabolism pathway, the Z4S treatment suppressed the generation of indole, thereby avoiding the accumulation of irritant odors. Tryptophan was stepwise metabolized into downstream products including tryptamine, serotonin, melatonin, and L-kynurenine. The abundances of tryptophan and its downstream products were highest in the Z4C group, indicating the strongest metabolic activity; they decreased in the Z4S and Z4G groups and reached the lowest levels in the Z4W group. This suggested that lowering the temperature and prolonging the duration of the stem-drying stage significantly blocked the cascade conversion of tryptophan to its downstream products, thus effectively reducing the formation of nitrogen-containing byproducts. In the tyrosine metabolism pathway, the Z4G treatment enhanced the decarboxylation of tyrosine to tyramine, which was further converted into salidroside, 4-hydroxyphenylacetate, and pyruvate. The relative content of salidroside was highest in the Z4G group; salidroside can be used as an exogenous flavor enhancer sprayed onto tobacco leaves to improve aroma quality. In the phenylalanine metabolism pathway, the transamination of phenylalanine to phenylpyruvate was suppressed in both the Z4G and Z4C treatments. In contrast, the conversion of phenylalanine to trans-3-hydroxycinnamate catalyzed by phenylalanine ammonia-lyase (*PAL*) exhibited higher abundance in the Z4C group, whereas this conversion was inhibited in the Z4G group.

## 4 Discussion

This study systematically compares the effects of different stage-specific curing processes on the quality and metabolite profiles of Yunyan 87 tobacco leaves of the C4F grade and reveals the potential mechanisms by which curing parameters influence leaf sensory quality through the regulation of key metabolic pathways. The results show that the Z4G treatment achieves the highest sensory score, which is likely associated with the lower starch and protein contents as well as the higher reducing sugar level observed in this group. Previous studies demonstrate that starch and protein contents are generally negatively correlated with tobacco aroma quality, whereas sugar content exhibits a positive correlation (Ye et al., 2024; Gong et al., 2023). The low-starch, high-sugar metabolic characteristics displayed by the Z4G group are mainly attributable to its optimized yellowing and color-fixing regimes. Studies report that elevated relative humidity not only promotes starch degradation but also accelerates chlorophyll breakdown by increasing chlorophyllase activity (Huang et al., 2026). This is consistent with the ultrastructural observations of chloroplasts in **Figure 1**. The degradation of starch granules is more thorough in the Z4G group than in other treatments, and soluble sugars accumulate in the Z4G group, as reflected in the starch and sucrose metabolism pathway (**Figure 6**). When the temperature exceeds 70 °C or the environment is under low humidity, the interaction between amylase and its substrate is inhibited (Zhao et al., 2026). Accordingly, the suitable temperature and high humidity maintained during the yellowing stage in this study sustain high hydrolytic activity toward starch granules, thereby facilitating the conversion of starch into small-molecule sugars (Banožić et al., 2020). In this process, starch is initially hydrolyzed by the combined action of endo-amylases and exo-amylases to yield maltose, dextrins, and other oligosaccharide products, which are subsequently subjected to debranching enzymes that hydrolyze the α-1,6 glycosidic linkages in amylopectin, thereby completely dismantling the starch backbone. Ultimately, through the sequential catalytic action of multiple enzymes, these intermediates are converted into soluble sugars (Zhao et al., 2026). Among various sugars, fructose and sucrose contribute most notably to sweetness (Wei et al., 2025b). Notably, the data reveal that the sucrose content in the Z4G group is significantly higher than that in the Z4C group. The elevated sucrose level may serve as the key material basis for the highest scores in aroma quantity and aroma quality of Z4C, since sucrose directly participates in the Maillard reaction during tobacco combustion (Celestino et al., 2024). Furthermore, the heatmap of DAMs involved in the starch and sucrose metabolism pathway shows that, except for Z4C, the relative contents of sugars such as D-glucose, cellobiose, trehalose-6P, and trehalose are higher in the other three treatment groups than in the Z4C group. Potassium and chlorine are core mineral indicators for evaluating the combustibility and industrial suitability of flue-cured tobacco; the potassium content of high-quality flue-cured tobacco generally needs to fall within the appropriate range of 2.0%–3.5%. In the present study, except for Z4C, all treatment groups meet this criterion for high-grade tobacco (Zhang et al., 2024).

**Figure 6:**
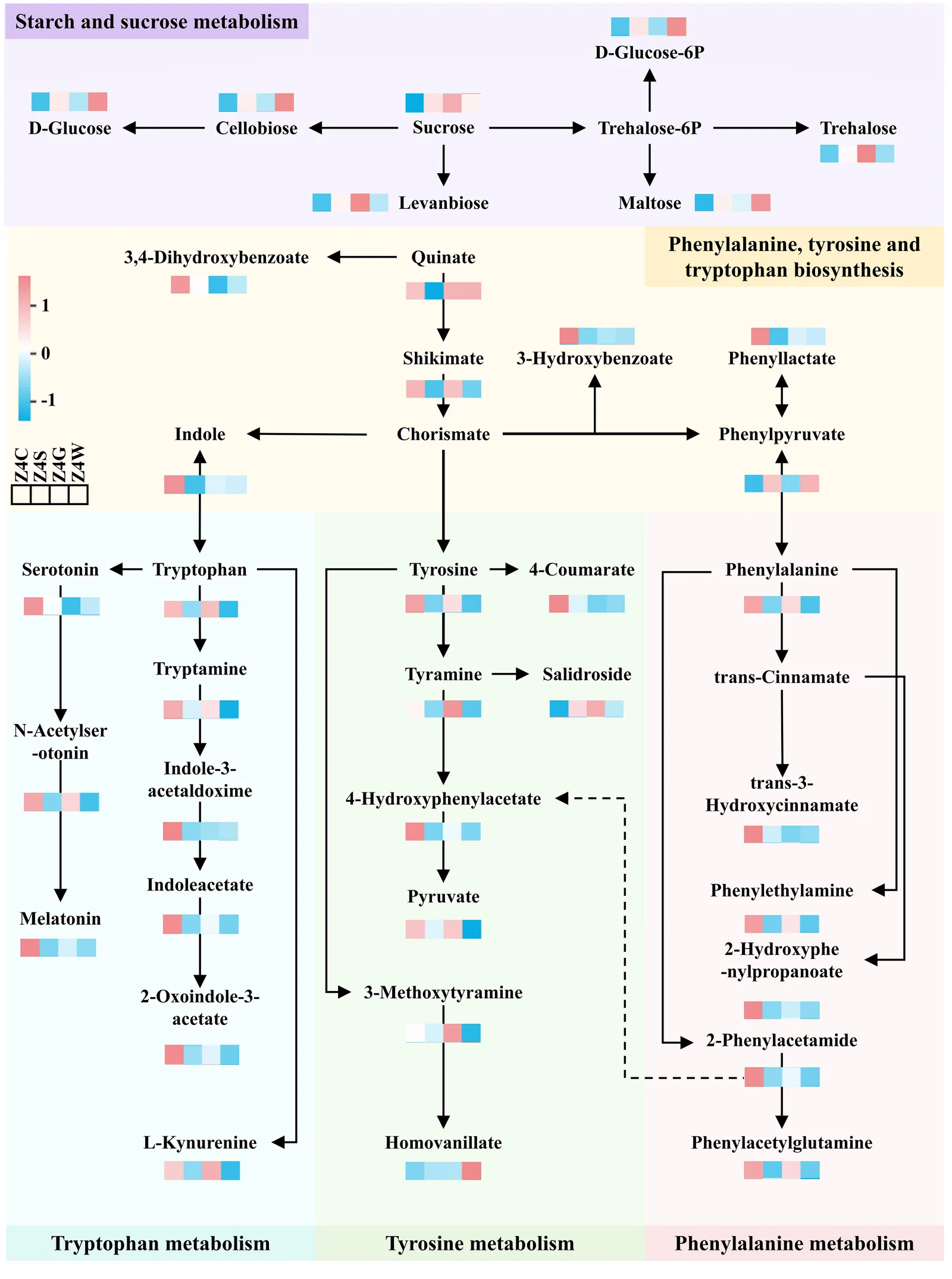
Metabolic pathway diagram: Starch and sucrose metabolism; Phenylalanine, tyrosine and tryptophan biosynthesis; Tryptophan metabolism; Tyrosine metabolism; Phenylalanine metabolism. Heatmaps are plotted based on the average peak areas of metabolites identified via LC-MS and GC-TOF-MS from five biological replicates per group. In the metabolite heatmaps embedded in the pathway, red indicates higher relative abundance, while blue represents lower relative abundance.

The Z4G and Z4W treatments reduce total alkaloid and total nitrogen contents while promoting the breakdown of proteins into free amino acids. However, the metabolomics data reveal a noteworthy concern: the Z4G treatment results in the highest level of NNK, a carcinogenic TSNA (Zhang et al., 2022). The conversion of nicotine to NNK is the result of synergistic effects between microbial activity and chemical reactions. During this process, nitrate is reduced to nitrite by microorganisms such as Bacillus, Staphylococcus, and Corynebacterium, while nicotine is oxidatively ring-opened to yield pseudooxynicotine, the direct precursor. Subsequent nitrosation of these two species ultimately produces NNK (Chopyk et al., 2017; Kumari et al., 2026). This explains why the Z4G group exhibits the lowest relative nicotine content yet the highest NNK level. This observation is likely attributable to the high relative humidity environment employed in the Z4G process, which alters microbial activity and denitrification dynamics, thereby promoting the continuous accumulation of TSNAs and nitrites and consequently accelerating the formation of nitrosamines such as NNK (Law et al., 2016). Thus, while optimizing curing parameters to enhance tobacco aroma quality, attention should also be paid to controlling TSNAs levels. Other TSNAs include N’-nitrosonornicotine (NNN), N’-nitrosoanabasine (NAB), and N’-nitrosoanatabine (NAT). During the curing process, nornicotine, anabasine, and anatabine react to generate NNN, NAT, and NAB, respectively (Zhao et al., 2026). In the present study, anatabine and anabasine are identified, and their relative contents, together with those of beta-nicotyrine, cytisinicline, and S-(-)-cotinine, all reach their lowest levels in the Z4W treatment. This outcome may be associated with the lower temperature applied during the stem-drying stage in the Z4W process, which likely reduces dry matter loss during this phase. Jia et al. (2023) report that increasing curing temperature during the stem-drying stage accelerates the volatilization and loss of free nicotine in tobacco leaves, ultimately leading to reduced contents of nicotine and total alkaloids in cured leaves. In the present study, lowering the curing temperature at the stem-drying phase retains nicotine and alkaloid levels, which mutually validates the above findings.

Certain flavonoids can generate aroma through pyrolysis during tobacco curing and combustion, while also neutralizing free radicals in smoke and reducing irritation (Wei et al., 2025a). The relative contents of the flavonoid compounds identified among all DAMs increase to varying extents across the three treatment groups, with the most pronounced elevation observed in the Z4W group, suggesting that a mild thermal environment helps to reduce thermal decomposition losses of flavonoids. Compared with the Z4C curing process, the Z4G curing process elevates temperature and humidity during the yellowing stage of tobacco leaf curing, which increases the relative abundance of flavonoids. Studies by Wei et al. (2025a) further verify that raising the wet-bulb temperature at the yellowing stage significantly upregulates the expression of key enzyme genes involved in flavonoid biosynthesis, namely *4-coumarate-CoA ligase* (*4CL*), *hydroxycinnamoyl-CoA shikimate/quinate hydroxycinnamoyl transferase* (*HCT*) and *flavanone 3-hydroxylase* (*FLS*). By contrast, temperature and humidity regulation at the color-fixing and stem-drying stages exerts negligible effects on the expression of these enzymes. Amino acids serve as core precursor substrates for the Maillard browning reaction, which generates a large number of characteristic aroma components during tobacco curing (Xu et al., 2025). The analysis reveals that the 28 differential amino acids exhibit the highest expression levels in the Z4C group, followed by the Z4G group, while lower expression levels are detected in the Z4S and Z4W groups. This trend is not entirely consistent with the changes in total amino acid content across groups, which may be attributed to variations in other amino acid species, as at least 117 amino acids are identified in different types of tobacco (Wang et al., 2020). Increasing temperature and humidity during the yellowing stage reduces the relative contents of aromatic amino acids, which is consistent with the findings reported by Wang et al. (2014). The temperature is maintained at approximately 65 °C during the stem-drying stage, and the intensity of the Maillard reaction increases with rising temperature (El Hosry et al., 2025). It is therefore speculated that tobacco leaves in the Z4W treatment are exposed to high temperature and high humidity for a longer duration, which significantly prolongs the period of the Maillard reaction throughout curing and leads to greater consumption of amino acid substrates. In contrast, the shorter color-fixing stage in the Z4S group restricts protein degradation. Furthermore, high temperatures upon entering the stem-drying stage further suppress the activity of proteolytic enzymes (Mozhaev and Martinek, 1982). Collectively, these factors result in lower levels of such amino acids in both the Z4S and Z4W treatments. Furthermore, the mapped pathways of tryptophan metabolism, tyrosine metabolism, and phenylalanine metabolism also reveal that the relative contents of the three aromatic amino acids and their metabolites are higher in the Z4C and Z4G groups.

Focusing on volatile aroma components, phytol, a core degradation product of chlorophyll, can further undergo dehydration to generate neophytadiene, which imparts fresh and hay-like notes and is the most abundant terpenoid in tobacco (Jiang et al., 2024; Wu et al., 2020). In the present study, the relative content of phytol is significantly higher in all three treatment groups than in the Z4C treatment, with the highest accumulation levels observed under the Z4S and Z4W processes. Geraniol, a monoterpene alcohol and a thermosensitive compound with a rose-like aroma (Chang et al., 2026), shows high accumulation in the Z4W group, indicating that a low-temperature environment during the stem-drying stage suppresses the volatilization loss and thermal oxidative degradation of such compounds, thereby preserving the fresh floral notes (Celebioglu et al., 2025). High retention of geraniol and phytol in the Z4W treatment correlates with the sensory scores including aroma quality and aftertaste, which further verifies that low-temperature drying facilitates the preservation of terpenoid aroma precursors and acts as a vital approach to optimizing the sensory quality of cured tobacco leaves. Benzyl alcohol and acetophenone are mainly generated from phenylalanine and tyrosine via multi-step thermal transformation in the Maillard reaction, and they can also be produced through direct thermal degradation of these two aromatic amino acids (Liu et al., 2026). The stem-drying temperature of the Z4C treatment is set at 68 °C, a condition that effectively facilitates the above thermal transformation and pyrolysis processes. Meanwhile, metabolomics data reveal a higher abundance of aromatic amino acids in this treatment. Combined, these two factors contribute to markedly elevated accumulation of benzyl alcohol and acetophenone in Z4C. These compounds endow tobacco leaves with floral, fruity and sweet aroma notes (Xu et al., 2024; Wu et al., 2024; Zhang et al., 2025).

KEGG pathway enrichment analysis in the present study demonstrates that the Z4G regime remodels carbohydrate metabolic networks in tobacco leaves via temperature-humidity modulation during the yellowing phase, facilitating concurrent accumulation of soluble saccharides including sucrose, trehalose and maltose. As pivotal precursor substrates fueling the Maillard cascade, the abundant buildup of soluble sugars furnishes sufficient metabolic precursors for generating desirable characteristic aromatic compounds (e.g., furans and pyrazines) upon cigarette combustion (Hao et al., 2025), which mechanistically elucidates the superior performance of Z4G group leaves in sensory indices of aroma quantity and aroma quality at the metabolic level. By comparison, the Z4S treatment induces pronounced depletion of shikimic acid and chorismic acid, two core intermediates of the shikimate pathway, which correspondingly constrains the synthetic flux of downstream aromatic amino acids. Aromatic amino acids exert dual physiological functions: they act as Maillard reaction substrates to synthesize floral and sweet odorants, while also serving as precursors for polyphenols and irritating off-flavor metabolites like indole via multi-stage metabolic transformations (Kisa et al., 2021). Repressed flux through this pathway diminishes the formation of indole-derived irritants in Z4S leaves yet concurrently depletes the pool of phenylpropanoid aroma precursors and restricts substrate availability for aroma biosynthesis; the superimposed effects of these two metabolic shifts plausibly account for the worst overall sensory performance observed in this treatment. With respect to tyrosine metabolism, the Z4G regime markedly upregulates the biosynthesis of salidroside, a characteristic aromatic glycoside, and the high abundance of this metabolite exhibits a strong positive correlation with the outstanding aroma quality of Z4G leaf samples. This observation corroborates that moderately elevated temperature and humidity throughout yellowing can selectively stimulate the enrichment of beneficial aroma glycosides, thereby ameliorating the smoke aromatic profiles of cured tobacco. Importantly, all leaf specimens deployed for the three treatments are pre-screened rigorously in compliance with the national tobacco grading standard GB 2635-1992, displaying highly uniform morphological and phenotypic characteristics. Nonetheless, integrated metabolomics profiling and sensory panel assessment clearly reveal that these phenotypically homogeneous leaves undergo profound divergence in endogenous chemical composition and ultimate sensory attributes following curing under disparate temperature-humidity protocols. The phenomenon of “uniform external morphology yet divergent internal metabolic profiles” essentially stems from divergent reprogramming of intracellular metabolic fluxes triggered by minor fluctuations in curing temperature and humidity. Slight modifications to these curing parameters are cascaded and amplified across the global metabolic network, and such profound metabolic discrepancies are ultimately manifested as distinguishable sensory defects across multiple evaluation dimensions, encompassing aroma quantity, aroma quality, sensory irritation and residual aftertaste.

Metabolomic differential analysis of tobacco leaf samples collected at the end of curing under different processes can directly reflect the ultimate impact of distinct curing processes on metabolite composition, aroma constituents, and the accumulation of harmful products. Nevertheless, this research still has three obvious limitations worthy of discussion: First, all field cultivation and curing experiments are only completed in a single production area in Lincang, Yunnan; regional soil, climate and curing equipment characteristics limit the generalizability of the research conclusions, and multi-site ecological trials are required for subsequent verification. Second, this study only carries out correlation analysis of differential metabolites based on omics data, without supporting functional experiments such as key enzyme activity determination and gene expression quantification, so it is impossible to confirm the causal regulatory relationship between curing parameters and metabolic changes. Third, we only make qualitative comparison of grouping trends between metabolites and sensory scores, and do not construct quantitative correlation models between metabolite abundance and multi-dimensional sensory evaluation indicators. Furthermore, tobacco curing is a continuous and dynamic physiological and biochemical process accompanied by water loss, enzymatic catalysis, and substance interconversion. Relying solely on single-point end-point samples cannot fully resolve the dynamic changes of metabolites across the entire curing cycle under different processes, making it difficult to pinpoint the core regulatory nodes and critical time windows where metabolic pathways undergo transitions and key precursors are synthesized. Future studies can perform time-series sampling at multiple points across key curing stages and employ time-resolved metabolomics to dissect the dynamic remodeling of metabolic networks and uncover the intervention points of the curing process, thereby more systematically elucidating the molecular mechanisms by which curing processes regulate the formation of intrinsic leaf quality.

## 5 Conclusion

This study integrated multi-platform metabolomics, physicochemical characterization and sensory evaluation to systematically dissect the metabolic mechanisms underlying quality formation of C4F-grade flue-cured tobacco leaves under staged curing regulation. The results demonstrated that curing parameters exerted significant modulatory effects on the intrinsic quality of tobacco leaves with consistent morphological appearances. The Z4G regime elevated temperature and humidity during the yellowing stage while lowering the wet-bulb temperature at the color-fixing phase, which facilitated thorough starch degradation and synchronous accumulation of soluble sugars, thereby improving aromatic performance and overall sensory quality. Nevertheless, this treatment also triggered elevated accumulation of NNK. Subsequent studies can use the Z4G process as a baseline to establish multi-gradient combination experiments of temperature and humidity during the yellowing stage, and systematically examine the effects of further changes in these parameters on the relative content of NNK. The Z4S protocol shortened the color-fixing period to cut total curing duration, elevated the abundances of aromatic components including cinnamic acid and phytol, and suppressed the biosynthesis of irritant indole; however, incomplete protein degradation resulted in the poorest comprehensive sensory quality and limited industrial applicability of leaves under this treatment. The Z4W treatment adopted prolonged low-temperature treatment in the stem-drying stage, which effectively mitigated the loss of thermolabile aroma precursors or volatile aroma components such as flavonoids, phytol and geraniol. On this basis, subsequent optimization could integrate the efficient sucrose accumulation strategy of Z4G during yellowing and the low-temperature protection scheme of Z4W in the stem-drying phase in a staged combined curing pattern, aiming to sustain superior aroma quality and further enhance sweet sensory notes, while also reducing the levels of NNK and other substances. This study provides a metabolomics theoretical basis for the precise staged flue-curing process of medium-grade C4F tobacco leaves, and it further reveals the vital importance of maintaining a dynamic balance between aroma improvement and harmful substance control throughout the tobacco curing process. In the future, intelligent prediction and online regulation systems tailored to the flue-curing process can be developed to translate the mechanistic findings of this study into engineering applications. For the first time, this study uncovered quality differentiation among visually uniform medium-grade tobacco leaves subjected to differentiated staged curing manipulations, which provides solid theoretical foundations and dataset support for precise optimization of curing protocols and improvement of industrial usability for C4F-grade and analogous tobacco leaf materials.

## Data Availability

The original contributions presented in the study are included in the article/**Supplementary Material**. Further inquiries can be directed to the corresponding authors.
