# Supplementary material for: LC-MS and GC-TOF-MS metabolomics elucidate quality and metabolite variations of medium-grade Yunyan 87 (C4F) tobacco under stage-specific curing process
Source: Front Plant Sci. 2026 Oct 30;17:1896995. doi: 10.3389/fpls.2026.1896995 (PMC13759782; doi:10.3389/fpls.2026.1896995)
Supplement: Supplementary file 1 [file DataSheet1.zip › Supplementary Materials/Supplementary materials.docx]

**LC-MS and GC-TOF-MS Metabolomics Elucidate Quality and Metabolite Variations of Medium-Grade Yunyan 87 (C4F) Tobacco Under Stage-Specific Curing Process**

**Meng Zhao^1,2^, Chaopeng Song^1^, Jiaen Su^1^,** **Jun He^4^, Mingdong He^4^, Meixun Fan^4^, Jiangtao Li^4^, Wei Yang^4^, Chunfeng Tao^4^, Hongwei Xiao^2,*^ Binbin Hu^1,3*^,**

^1^ Yunnan Academy of Tobacco Agricultural Sciences, Kunming 650021, China;

^2^ College of Engineering, China Agricultural University, Beijing, 100083, China;

^3^Yunnan Daguan Laboratory, Kunming 650021, China;

^4^Lincang City Tobacco Monopoly Bureau, Lincang, 677099, China;

*** Correspondence:**

Binbin Hu (hubinbin20072008@163.com)
Hongwei [Xiao (xhwcaugxy@163.com)]("mailto:Xiao)

**Supplementary Figures 1-4**

Figure S1 A, Overlapping total ion current (TIC) chromatograms of QC samples in negative ion mode by LC-MS; B, Overlapping TIC chromatograms of QC samples in positive ion mode by LC-MS; C, Pearson correlation analysis of QC samples from LC-MS; D, Pearson correlation analysis of QC samples from GC-TOF-MS.

Figure S2 PLS-DA score scatter plots and permutation validation plots based on LC-MS results (200 response permutations). Each color corresponds to an independent curing treatment group. In the score scatter plots, R2Y represents the explanatory rate of the model, and Q2Y is used to evaluate the predictive ability of the model; a model is well-established when R2Y is greater than Q2Y. In the permutation validation plots, the model is not overfitted when the R2 data are greater than the Q2 data and the intercept of the Q2 regression line with the Y-axis is less than 0.

Figure S3 PLS-DA score scatter plots and permutation validation plots based on GC-TOF-MS results (200 response permutations). Each color corresponds to an independent curing treatment group. In the score scatter plots, R2Y represents the explanatory rate of the model, and Q2Y is used to evaluate the predictive ability of the model; a model is well-established when R2Y is greater than Q2Y. In the permutation validation plots, the model shows no overfitting when the R2 values are greater than the Q2 values and the intercept of the Q2 regression line with the Y-axis is less than 0.

Figure S4. Correlation chord diagrams of flavonoids, terpenoids and their derivatives, amino acids, and fatty acids across comparison groups. Pearson correlation analysis was performed on log₂-transformed data with screening thresholds set at |r| > 0.9 and P < 0.05. Different colors correspond to different classes of metabolites, and the color gradient of connecting lines in the middle represents the magnitude of correlation coefficients. Four metabolite categories plotted in the diagram include flavonoids, terpenoid derivatives, amino acids, and fatty acids.
